# Personal Exposure to Radio Frequency Electromagnetic Fields among Australian Adults

**DOI:** 10.3390/ijerph15102234

**Published:** 2018-10-12

**Authors:** Berihun M. Zeleke, Christopher Brzozek, Chhavi Raj Bhatt, Michael J. Abramson, Rodney J. Croft, Frederik Freudenstein, Peter Wiedemann, Geza Benke

**Affiliations:** 1 Centre for Population Health Research on Electromagnetic Energy (PRESEE), School of Public Health and Preventive Medicine, Monash University, Melbourne, VIC 3004, Australia; christopher.brzozek@monash.edu (C.B.); michael.abramson@monash.edu (M.J.A.); geza.benke@monash.edu (G.B.); 2 Department of Epidemiology and Preventive Medicine, School of Public Health and Preventive Medicine, Monash University, 553 St Kilda Road, Melbourne, VIC 3004, Australia; chhavi.bhatt@monash.edu; 3 Monash University Endocrine Surgery Unit, Alfred Hospital, 55 Commercial Rd, Melbourne, VIC 3004, Australia; 4 Monash Emergency Service, Monash Health, Dandenong Hospital, 135 David Street, Melbourne, VIC 3175, Australia; 5 Australian Centre for Electromagnetic Bioeffects Research, Illawarra Health and Medical Research Institute, School of Psychology, University of Wollongong, Northfields Ave, Wollongong, NSW 2522, Australia; rcroft@uow.edu.au (R.J.C.); frederik@uow.edu.au (F.F.); peter.wiedemann@wf-emf.org (P.W.)

**Keywords:** radiofrequency electromagnetic fields, personal exposure measurement, mobile phone base stations, downlink, uplink

## Abstract

The measurement of personal exposure to radiofrequency electromagnetic fields (RF-EMFs) is important for epidemiological studies. RF-EMF exposure can be measured using personal exposimeters that register RF-EMFs over a wide range of frequency bands. This study aimed to measure and describe personal RF-EMF exposure levels from a wide range of frequency bands. Measurements were recorded from 63 participants over an average of 27.4 (±4.5) hours. RF-EMF exposure levels were computed for each frequency band, as well as from downlink (RF from mobile phone base station), uplink (RF from mobile phone handsets), broadcast, and Wi-Fi. Participants had a mean (±SD) age of 36.9 ± 12.5 years; 66.7% were women; and almost all (98.2%) from urban areas. A Wi-Fi router at home was reported by 61 participants (96.8%), with 38 (61.2%) having a Wi-Fi enabled smart TV. Overall, 26 (41.3%) participants had noticed the existence of a mobile phone base station in their neighborhood. On average, participants estimated the distance between the base station and their usual residence to be about 500 m. The median personal RF-EMF exposure was 208 mV/m. Downlink contributed 40.4% of the total RF-EMF exposure, followed by broadcast (22.4%), uplink (17.3%), and Wi-Fi (15.9%). RF-EMF exposure levels on weekdays were higher than weekends (*p* < 0.05). Downlink and broadcast are the main contributors to total RF-EMF personal exposure. Personal RF-EMF exposure levels vary according to day of the week and time of day.

## 1. Introduction

In recent decades, people are being increasingly exposed to man-made radiofrequency-electromagnetic fields (RF-EMFs). These fields range between 0.1 and 6 GHz frequency bands and are classified into two broad categories: near-field (mobile phones, iPads, tablets, laptops, and so on), and far-field sources (mobile phone base stations, Wi-Fi routers, radio/television broadcasting towers, mobile phones in the surroundings, and so on) [1]. Near-field sources operate in close proximity to the body and result in non-uniform RF-EMF exposure, while far-field sources, which operate from far greater distances from the body and typically result in a much lower but more uniform level of RF-EMF exposure [2,3].

Personal RF-EMF exposure assessment has been a challenging task in human epidemiological studies. However, personal RF-EMF exposure to a wide range of frequency bands, including mobile phone base stations, Wi-Fi networks, FM radio, etc. can be measured using personal exposimeters [4,5]. The World Health Organization (WHO) prioritized research into understanding the health effects of RF-EMF emphasizing the need to measure personal exposures [6]. Subsequently, measurement protocols have been suggested and implemented by researchers [7,8,9].

Previous studies in Europe have reported personal RF-EMF exposures using exposimeters [9,10,11] although most were limited to predefined micro-environment measurements over a short time or did not address the issue of personal RF-EMF exposures [7,8,12,13]. Such measurements are known to introduce significant variability according to the type and location of the selected micro-environments, the RF-EMF frequency band considered, and the time of day measurements were performed [12,13]. Although some studies found that exposure to RF-EMF can depend on many complex factors, exposimeters can help capture different sources of personal RF-EMF exposure, and evaluate how this exposure varies over time [4,9,14,15]. It is therefore important to generate more local data on personal RF-EMF exposures from common sources of RF in the environment.

In this study, we conducted RF-EMF exposure measurements among Australian adults using personal exposure measurement meters (ExpoM-RF) for approximately 24 consecutive hours. The purposes of this study were to: (1) evaluate personal RF-EMF exposure levels without limiting them to specific micro-environments, so as to assess an individual’s level of exposure in a routine 24 h period, and (2) describe the RF-EMF exposure from each frequency band over the whole measurement period by occupation category, time of the day, and day of the week.

## 2. Materials and Methods

### 2.1. Study Design and Participant Recruitment

This was part of a study investigating the effect of providing objectively measured RF-EMF exposure levels on the risk perception of people towards the potential health effects of RF-EMF exposure from mobile phone base stations and Wi-Fi routers. Between June and November 2017, participants were invited to participate in an experimental study via advertisements posted on notice boards at public libraries, sporting clubs, universities, and hospitals across Melbourne, Australia. Participants were then given a plain language information pack detailing the study procedures, and consent forms. After written informed consent was obtained, participants allocated to the measurement group were then provided with personal RF-EMF measurement devices. This paper reports the detailed findings from the personal measurement group of the experimental study. The study was approved by the Monash University Human Research Ethics Committee (MUHREC 8965, 22 May 2017).

### 2.2. Personal Exposure Measurement Devices

**Figure 1:**
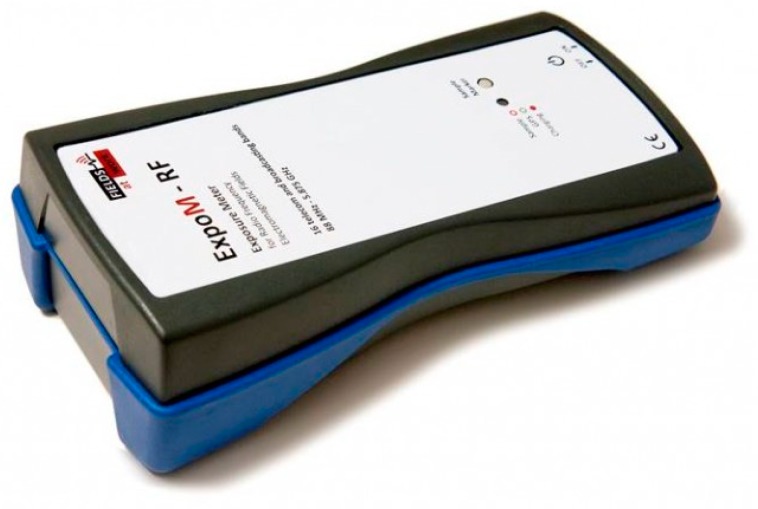
ExpoM-RF (Fields at Work GmbH, Zürich, Switzerland).

Personal RF-EMF exposure measurements were performed between June and November 2017, using ExpoM-RF devices (Zürich, Switzerland) (Fields At Work [16]. Each participant carried an exposimeter (approximate weight 320 g and dimensions 16 × 8 × 4 cm, see Figure 1) in a small hip bag for approximately 24 consecutive hours. When receiving the device, participants were also given detailed written instructions regarding personal measurements. Participants were instructed in person and in detail about how to handle the exposimeter during the measurements. Participants were asked to continue their usual daily activities while wearing the ExpoM-RF, but to place it on their bedside table or close to their bed when asleep. The ExpoM-RF measures electric field strengths between 0.005 and 5 V/m in 16 different RF-EMF frequency bands. Measurement intervals were adjusted to 10 s.

The exposure levels in root mean square (RMS in V/m units) were collected from the 16 different RF-EMF frequency bands (87.5 MHz–5.8 GHz). Total RF-EMF referred to the sum of all measured frequency bands except, WiMax 3.5 GHz and ISM 5.8 GHz. We excluded these frequencies from further analysis because of crosstalk concerns with other bands and their inclusion would overestimate the total exposure [1]. All other frequency bands were further computed and summarized into five main groups: (i) downlink (DL; RF-EMF exposure from mobile phone base station exposure) exposure—RMS sum of all downlink frequency bands (LTE 800 MHz, GSM 900 MHz, GSM 1800 MHz, UMTS 2100 MHz and LTE 2600 MHz), (ii) uplink (UL; RF-EMF exposure from mobile phone handsets) exposure—RMS sum of all uplinks (LTE 800 MHz, GSM 900 MHz, GSM 1800 MHz, UMTS 2100 MHz, and LTE 2600 MHz), (iii) Wi-Fi (ISM 2.4 GHz), (iv) digital enhanced cordless telecommunications (DECT), and (v) broadcast—sum of FM Radio and DVB-T (TV) frequencies.

Exposure levels from all broadband and narrow frequency bands were summed and expressed as a percentage of the total RF-EMF personal exposure levels. However, the devices we used do not specify if the measured RF-EMF exposure was from the participant’s own cell phone or from other people’s mobile phone use. We report electric field strength values (in mV/m) in the results section, since that is most commonly reported in the literature [2,7,17,18]. We also compared the exposures with the International Commission on Non-Ionizing Radiation Protection (ICNIRP) reference levels for common frequency bands [19].

### 2.3. Statistical Analysis

To describe RF-EMF exposure from each frequency band over the whole measurement period as a function of time of the day, day of the week and occupation group, we calculated mean and median exposures, as well as other summary statistics. Each 24-h period was divided into 4 × 6-h intervals (from 00:00–06:00, 06:00–12:00, 12:00–18:00 and 18:00–00:00); and 2 intervals each for daytime (06:00–18:00) and night-time (18:00–06:00). The days of the week on which measurements were performed were summarized into two groups (weekday or weekend). Exposure values for each participant were averaged for each of the time slots, as well as, day- and night-time intervals separately.

We also calculated the percentage contributions of each main group of bands: downlink (exposure from mobile phone base stations), uplink (exposure from mobile phone handsets), broadcast (exposure from FM radio and TV), and Wi-Fi to the total RF-EMF exposure (sum of downlink, uplink, broadcast and Wi-Fi) as a function of time slots of the day, and days of the week (weekday vs weekend).

Normality was tested for each main frequency band. We present the median values since the exposure distributions were not normal. Kruskal–Wallis tests were performed to compare median RF-EMF levels by predefined variables such as gender, occupation, days of the week (weekend vs. weekday) and time of the day. For all statistical tests performed, *p*-values < 0.05 were considered statistically significant. All statistical analyses were carried out using STATA (version 14, StataCorp, College Station, TX, USA).

## 3. Results

### 3.1. Participant Profile

Personal RF-EMF exposure measurements were conducted of 63 adults, aged between 18 and 72 years (mean ± SD age of 36.9 ± 12.5 years and 66.7% women). Almost all (98.2%) lived in metropolitan areas. A Wi-Fi router at home was reported by 61 participants (96.8%), with 38 (61.2%) having a Wi-Fi enabled smart TV. A mobile phone base station in the neighborhood was noticed by 26 (41.3%) participants. The average estimated distance of the closest mobile phone tower of which the participant was aware of was 500 m.

Overall, 17 (27%) were working in the healthcare sector, 16 (25.4%) in office support and administration, 10 (15.9%) were researchers or university teachers, 10 (15.9%) were working in the education sector or studying (school teacher, primary/childcare educator or student), while the rest (*n* = 10; 15.9%) were from other sectors such as engineering, food and hospitality services (Table 1).

### 3.2. RF-EMF Exposure Levels

On average, 9764 (range: 7236–13,536) single measurements per participant were recorded over an average measurement time of 27.4 ± 4.5 h (range: 20.1–37.6). Participants carried the ExpoM-RF including time spent inside and outside the home, both during day- and night-time. Measurements for 45 (71.4%) participants were performed over weekdays and on weekends for the remaining 18 (28.6%).

Table 2 presents the summary statistics (mean, median, 25th, 75th, and 99th percentiles) of personal RF-EMF exposures for each frequency band. The three highest personal RF-EMF exposures and their contributing sources were: GSM 1800 MHz DL (29 mV/m); GSM 900 MHz UL (24 mV/m); and DVB-T (24 mV/m). The median personal RF-EMF exposure of all participants was total 208 mV/m, downlink 87 mV/m, uplink 37 mV/m, broadcast 48 mV/m, and Wi-Fi 2.4 GHz 23 mV/m (Table 2).

**Figure 2:**
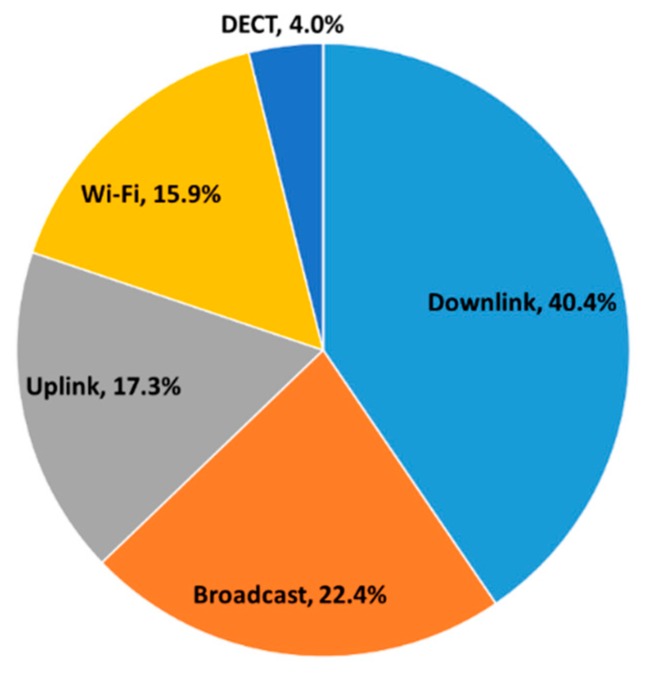
Percentage contributions of each general frequency band medians to the median total RF-EMF exposure over the measurement period for all participants (n = 63). Uplink = LTE 800 MHz UL + GSM 900 MHz UL + GSM 1800 MHz UL + UMTS 2100 MHz UL + LTE 2600 MHz UL; Downlink = LTE 800 MHz DL + GSM 900 MHz DL + GSM 1800 MHz DL + UMTS 2100 MHz DL + LTE 2600 MHz DL; Wi-Fi (Wireless Fidelity) = ISM 2400 GHz; Broadcast = FM Radio + DVB-T (TV); DECT, Digital-Enhanced Cordless Telecommunications. Total RF-EMF was calculated as the sum of all measured frequency bands except, WiMax 3.5 GHz and ISM 5.8 GHz.

The overall percentage contribution of RF-EMF sources of exposure to the total RF-EMF is presented in Figure 2. Downlink was the largest contributor to total RF-EMF exposure (40.4%) followed by broadcast (22.3%), uplink (17.4%), and Wi-Fi (15.9%). Overall, median RF-EMF exposure levels were in order of less than one-percent of the International Commission on Non-Ionizing Radiation Protection (ICNIRP) guideline limits for the general population.

**Figure 3:**
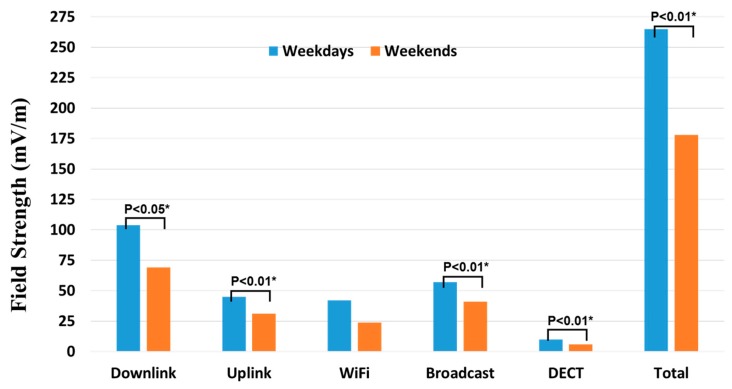
Distribution of median RF-EMF exposure over the measurement period, further segregated by weekdays and weekends (* Kruskal–Wallis tests). Uplink = LTE 800 MHz UL + GSM 900 MHz UL + GSM 1800 MHz UL + UMTS 2100 MHz UL + LTE 2600 MHz UL; Downlink = LTE 800 MHz DL + GSM 900 MHz DL + GSM 1800 MHz DL + UMTS 2100 MHz DL + LTE 2600 MHz DL; Wi-Fi (Wireless Fidelity) = ISM 2400 GHz; Broadcast = FM Radio + DVB-T (TV); DECT, Digital-Enhanced Cordless Telecommunications.

**Figure 4:**
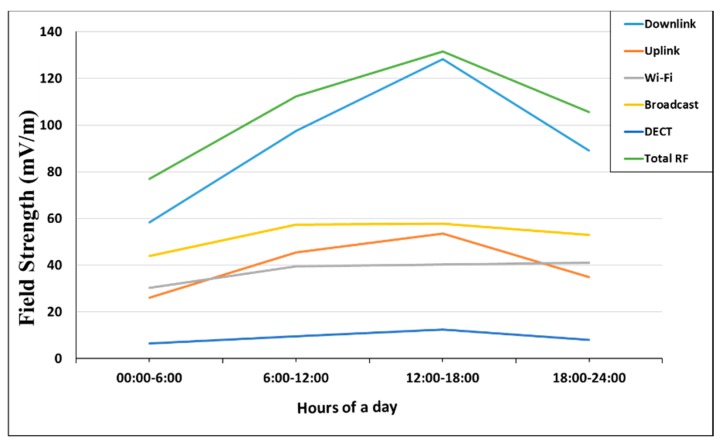
Median RF-EMF personal exposures from each general frequency band (uplink, downlink, Wi-Fi, broadcast, DECT, and total RF-EMF) by time slots of the day.

The median total RF-EMF exposure was higher on weekdays than weekends (233 vs. 162 mV/m; *p* = 0.003). Similarly, median RF-EMF exposures from downlink (93 vs. 56 mV/m; *p* = 0.025), uplink (50 vs. 28 mV/m; *p* = 0.006), and broadcast (50 vs. 33 mV/m; *p* = 0.002) were significantly higher during weekdays compared to that of weekends (Figure 3). Although the RF-EMF exposure from Wi-Fi was higher on weekdays compared with that for weekends, this difference was not significantly significant (*p* = 0.194). Comparing the medians for each of the time slots in the day demonstrated that exposures from downlink, uplink, as well as total RF-EMF showed an increasing trend in the first three time slots (from 00:00–06:00, 06:00–12:00, and 12:00–18:00) and then decreased in the evening (18:00–00:00). Subsequently, the median RF-EMF exposures levels from downlink and uplink sources were higher than that of night-time (*p* < 0.0001). No such trend was observed for Wi-Fi or broadcasting (Figure 4). Overall, adjusting for other factors including age, sex, and occupational category of participants did not show any significant effects.

## 4. Discussion

This RF-EMF personal exposure assessment study among Australian adults (aged 18 to 72 years) performed measurements over a routine 24 h period from all sources of RF-EMF without limitation to specific micro-environments. Our study provided comprehensive personal exposure measurements and compared RF-EMF exposure contributions of different sources as a function of time of day, and day of the week. This study was not limited to only mobile phone downlink signals, but presented detailed measurements for all frequency bands ranging between 87.5 MHz–5.8GHz that also included frequencies from Wi-Fi routers and broadcasting.

RF-EMF personal exposures from downlink and broadcast sources, followed by uplink and Wi-Fi, contributed the largest proportion to the total RF-EMF personal exposures. This was consistent with previous studies that identified downlink (from mobile phone base stations) and broadcast to be major sources of RF-EMF exposure [2,17,20] more than that from Wi-Fi routers [5,21]. RF-EMF exposure data from Australian children and adolescents also showed that typical RF-EMF levels were higher for broadcast and mobile phone base stations than Wi-Fi and DECT [1,22,23]. We also noted that the total RF-EMF personal exposure was higher on weekdays compared to weekends. Similarly, previous studies of RF-EMF exposure levels measured from various sources in different urban settings also reported higher RF-EMF exposure on weekdays compared to weekends [5,21].

Personal measurements are likely to more closely represent true exposure by investigating the exposure during all activities of everyday life of individuals. The measurement of RF-EMF exposures in the daily lives of people without restriction to specific micro-environments or specific time of the day enabled us to describe variations in RF exposure over a 24 h period and patterns that were slightly different over the days of the week and time of day. In addition, the RF-EMF exposimeters used are one of the best current tools for personal RF-EMF exposure [1].

Similar studies performed previously also reported a workday-weekday contrast and also difference between days of the week [14,24,25]. A more recent study in Melbourne performed micro-environment measurements for a duration of 15–20 min during the daytime and revealed variations of personal RF-EMF exposure depending on the time of the day, in which the morning resulted in lower exposure values in all studied exposure groups, except broadcasting [13].

Previous research [8,13] demonstrated high variability across the same micro-environment on different days and hours of the day in Australia. Nonetheless, the relatively smaller number of measurements for each day of the week and the lack of repeated measurements made it difficult to appreciate daily variations in the current study. Measured RF-EMF values depend highly on the distance between the emitting source and the measurement device, which is not necessarily the same as the distance between the emitting source and the body [4,9,15,26]. In the current study, distance from mobile phone base stations in the participant’s neighborhood was only a subjective estimation and did not assess the position of the mobile telephony antenna relative to the participant’s usual residence. Therefore, results are not directly comparable to previous studies that objectively assessed the height, distance, and direction of the closest mobile telephony antenna [26]. Although we instructed the participants in person and in detail about how to handle the exposimeter during the measurements, we were not able to control the positioning of the exposimeters during the personal measurements.

In addition, limitations linked to personal measurements such as inability to control for day-to-day variations in personal RF-EMF exposure [27], being a single measurement (cross-sectional nature), and inability to account for dependency in positioning/carrying of dosimeters were not accounted in this study.

Likewise, interference of measurements by the body (body shielding) may also contribute to potential underestimation depending on the frequency band of the RF-EMF source [19]. The effect of body shielding has previously been reported to give rise to measurement uncertainties as different positions and measurement environments give different responses to the same exposure [4,28,29,30]. Measurements were performed without limiting them to either indoor or outdoor environments although human RF-EMF exposure is known to vary due to multipath rays mainly in outdoor environments [31]. Furthermore, the inability to detect signals below the lower detection limits [2,8,20], and the exclusion of frequencies bands known to have crosstalk between neighboring frequencies (where power emitted in one frequency band is measured and reported in another band) may underestimate the total RF-EMF exposure [1,2]. Although we are aware that the use of one’s own cell phone is a large contributor to personal RF-EMF exposures, the exposimeters used in this study were not able to differentiate between RF exposure from one’s own mobile phones use and other people’s mobile phone use [10,32]. Since we did not collect data about mobile phone use patterns during the measurement periods, estimation of the contribution of uplink exposure from other people’s mobile phones was not possible in this study. Body calibration of the ExpoM-RF were not performed in this study, since we were interested in comparing our results to other studies where such calibrations were not usually conducted.

## 5. Conclusions

Downlink and broadcast, followed by uplink and Wi-Fi, were the main sources of RF-EMF personal exposure in this sample. Furthermore, personal RF-EMF exposure varied by days of the week and time slots over the day. However, reassuringly, the measurements suggested that all personal RF-EMF exposure levels were substantially below the ICNIRP guideline limits for the general population for RF frequency bands between 3 kHz and 300 GHz.

## Figures and Tables

**Table 1:** Socio-demographic characteristics of participants.

Characteristics	*n* (%)
Age, mean ± SD (years)	36.9 ± 12.5
18–24 years	8 (12.7)
25–34 years	26 (41.3)
35–44 years	14 (22.2)
45+ years	15 (23.8)
Sex	
Female	42 (66.7)
Race/Ethnicity	
Caucasian	33 (52.4)
Asian	23 (36.5)
Other	7 (11.1)
Residential location (*n* = 56)	
Metropolitan	55 (98.2)
Education	
High school or less	25 (39.7)
Beyond high school	38 (60.3)
Occupation	
Office support/admin/finance	16(25.5)
Healthcare	17(27.1)
Research	10(15.8)
Education sector	10(15.8)
Other	10(15.8)
Noticed a base station in the vicinity	
Yes	26 (41.3)
No	22 (34.9)
Not sure	15 (23.8)
Base station distance from usual residence (median; meters)	600
Have a Wi-Fi router at home	
Yes	61(96.8)
Smart TV connected to Wi-Fi in house	
Yes	38(61.3)

**Table 2:** Average personal exposure levels of all participants (*n* = 63) across different radiofrequency electromagnetic fields (RF-EMF) frequency bands (mV/m).

Frequency Bands	Mean	Median	25th, 75th, 99th Percentiles	Percent of Median Total RF-EMF (%)
FM Radio	23.1	18	15, 26, 110	8.4
DVB-T (TV)	29.0	24	14, 40, 91	11.2
LTE 800 MHz DL	6.7	6	5, 7, 22	2.8
LTE 800 MHz UL	6.2	5	4, 6, 18	2.3
GSM 900 MHz DL	28.4	22	15, 33, 147	10.2
GSM 900 MHz UL	26.0	24	12, 35, 72	11.2
GSM 1800 MHz DL	32.5	29	18, 41, 104	13.5
GSM 1800 MHz UL	4.1	3	2, 5,13	1.4
DECT	9.2	8	5, 12, 35	3.7
UMTS 2100 MHz DL	18.9	16	9, 23, 83	7.4
UMTS 2100 MHz UL	2.4	2	1, 3, 10	0.9
ISM 2.4 GHz (WLAN)	37.1	23	15, 35, 62	10.7
LTE 2600 MHz DL	7.7	5	3, 11, 40	2.3
LTE 2600 MHz UL	1.7	2	1, 2, 10	0.9
WiMax 3.5 GHz	1.4	1	1, 1, 2	0.5
ISM 5.8 GHz	5.6	3	1, 7, 38	1.4
Total ^a^	233.3	215	158, 262, 720	100.0

^a^ Sum of all enlisted frequency bands. Abbreviations: DECT, Digital-Enhanced Cordless Telecommunications; DL, downlink; DVB-T, Digital Video Broadcasting-Terrestrial; FM, Frequency Modulation; GSM, Global System for Mobile Communications; ISM, Industrial, Scientific and Medical; LTE, Long-Term Evolution; RF- EMF, radiofrequency-electromagnetic field; UL, uplink; UMTS, Universal Mobile Telecommunications System; WiMAX, Worldwide Interoperability for Microwave Access.

## References

[1] Roser K., Schoeni A., Struchen B., Zahner M., Eeftens M., Frohlich J., Röösli M. (2017). Personal radiofrequency electromagnetic field exposure measurements in Swiss adolescents. Environ. Int..

[2] Bolte J.F., Eikelboom T. (2012). Personal radiofrequency electromagnetic field measurements in The Netherlands: Exposure level and variability for everyday activities, times of day and types of area. Environ. Int..

[3] Martens A.L., Slottje P., Meima M.Y., Beekhuizen J., Timmermans D., Kromhout H., Smid T., Vermeulen R.C.H. (2016). Residential exposure to RF-EMF from mobile phone base stations: Model predictions versus personal and home measurements. Sci. Total Environ..

[4] Bolte J.F.B. (2016). Lessons learnt on biases and uncertainties in personal exposure measurement surveys of radiofrequency electromagnetic fields with exposimeters. Environ. Int..

[5] Joseph W., Frei P., Roosli M., Thuroczy G., Gajsek P., Trcek T., Bolte J., Vermeeren G., Mohler E., Juhász P. (2010). Comparison of personal radio frequency electromagnetic field exposure in different urban areas across Europe. Environ. Res..

[6] Van Deventer E., van Rongen E., Saunders R. (2011). WHO research agenda for radiofrequency fields. Bioelectromagnetics.

[7] Bhatt C., Thielens A., Billah B., Redmayne M., Abramson M.J., Sim M.R., Vermeulen R., Martens L., Joseph W., Benke G. (2016). Assessment of personal exposure from radiofrequency-electromagnetic fields in Australia and Belgium using on-body calibrated exposimeters. Environ. Res..

[8] Bhatt C.R., Thielens A., Redmayne M., Abramson M.J., Billah B., Sim M.R., Vermeulen R., Martens L., Joseph W., Benke G. (2016). Measuring personal exposure from 900 MHz mobile phone base stations in Australia and Belgium using a novel personal distributed exposimeter. Environ. Int..

[9] Roosli M., Frei P., Bolte J., Neubauer G., Cardis E., Feychting M., Gajsek P., Heinrich S., Joseph W., Mann S. (2010). Conduct of a personal radiofrequency electromagnetic field measurement study: Proposed study protocol. Environ. Health.

[10] Durrenberger G., Frohlich J., Roosli M., Mattsson M.O. (2014). EMF monitoring-concepts, activities, gaps and options. Int. J. Environ. Res. Public Health.

[11] Joseph W., Verloock L. (2010). Influence of mobile phone traffic on base station exposure of the general public. Health Phys..

[12] Sagar S., Adem S.M., Struchen B., Loughran S.P., Brunjes M.E., Arangua L., Dalvie M.A., Croft R.J., Jerrett M., Moskowitz J.M. (2018). Comparison of radiofrequency electromagnetic field exposure levels in different everyday microenvironments in an international context. Environ. Int..

[13] Thielens A., van den Bossche M., Brzozek C., Bhatt C.R., Abramson M.J., Benke G., Martens L., Joseph W. (2018). Representativeness and repeatability of microenvironmental personal and head exposures to radio-frequency electromagnetic fields. Environ. Res..

[14] FieldsatWork GmbH, Sonneggstrasse 60, 8006 Zürich, Switzerland. www.fieldsatwork.ch.

[15] Urbinello D., Joseph W., Verloock L., Martens L., Roosli M. (2014). Temporal trends of radio-frequency electromagnetic field (RF-EMF) exposure in everyday environments across European cities. Environ. Res..

[16] Urbinello D., Joseph W., Huss A., Verloock L., Beekhuizen J., Vermeulen R., Martens L., Röösli M. (2014). Radio-frequency electromagnetic field (RF-EMF) exposure levels in different European outdoor urban environments in comparison with regulatory limits. Environ. Int..

[17] ICNIRP (2009). ICNIRP statement on the “Guidelines for limiting exposure to time-varying electric, magnetic, and electromagnetic fields (up to 300 GHz)”. Health Phys..

[18] Gajsek P., Ravazzani P., Wiart J., Grellier J., Samaras T., Thuroczy G. (2015). Electromagnetic field exposure assessment in Europe radiofrequency fields (10 MHz-6 GHz). J. Expo. Sci. Environ. Epidemiol..

[19] Birks L.E., Struchen B., Eeftens M., van Wel L., Huss A., Gajsek P., Kheifets L., Gallastegi M., Dalmau-Bueno A., Estarlich M. (2018). Spatial and temporal variability of personal environmental exposure to radio frequency electromagnetic fields in children in Europe. Environ. Int..

[20] Karipidis K., Henderson S., Wijayasinghe D., Tjong L., Tinker R. (2017). Exposure to radiofrequency electromagnetic fields from Wi-Fi in Australian schools. Radiat. Prot. Dosimetry.

[21] Bhatt C.R., Redmayne M., Billah B., Abramson M.J., Benke G. (2017). Radiofrequency-electromagnetic field exposures in kindergarten children. J. Expo. Sci. Environ. Epidemiol..

[22] Frei P., Mohler E., Neubauer G., Theis G., Burgi A., Frohlich J., Braun-Fahrländer C., Bolte J., Egger M., Röösli M. (2009). Temporal and spatial variability of personal exposure to radio frequency electromagnetic fields. Environ. Res..

[23] Viel J.F., Cardis E., Moissonnier M., de Seze R., Hours M. (2009). Radiofrequency exposure in the French general population: Band, time, location and activity variability. Environ. Int..

[24] Viel J.F., Tiv M., Moissonnier M., Cardis E., Hours M. (2011). Variability of radiofrequency exposure across days of the week: A population-based study. Environ. Res..

[25] Buckus R., Strukcinskiene B., Raistenskis J., Stukas R., Sidlauskiene A., Cerkauskiene R., Isopescu D.N., Stabryla J., Cretescu I. (2017). A Technical Approach to the Evaluation of Radiofrequency Radiation Emissions from Mobile Telephony Base Stations. Int. J. Environ. Res. Public Health.

[26] Inyang I., Benke G., McKenzie R., Abramson M. (2008). Comparison of measuring instruments for radiofrequency radiation from mobile telephones in epidemiological studies: Implications for exposure assessment. J. Expo. Sci. Environ. Epidemiol..

[27] Aerts S., Wiart J., Martens L., Joseph W. (2018). Assessment of long-term spatio-temporal radiofrequency electromagnetic field exposure. Environ. Res..

[28] Bhatt C.R., Redmayne M., Abramson M.J., Benke G. (2016). Instruments to assess and measure personal and environmental radiofrequency-electromagnetic field exposures. Australas Phys. Eng. Sci. Med..

[29] De Miguel-Bilbao S., Blas J., Ramos V. (2018). Effective Analysis of Human Exposure Conditions with Body-worn Dosimeters in the 2.4 GHz Band. J. Vis. Exp..

[30] De Miguel-Bilbao S., Ramos V., Blas J. (2017). Responses to comments on assessment of polarization dependence of body shadow effect on dosimetry measurements in the 2.4 GHz band. Bioelectromagnetics.

[31] Rodriguez B., Blas J., Lorenzo R.M., Fernandez P., Abril E.J. (2011). Statistical perturbations in personal exposure meters caused by the human body in dynamic outdoor environments. Bioelectromagnetics.

[32] Sagar S., Struchen B., Finta V., Eeftens M., Roosli M. (2016). Use of portable exposimeters to monitor radiofrequency electromagnetic field exposure in the everyday environment. Environ. Res..

